# ELMO3 – a Negative Prognostic Marker in Minor Salivary Gland Carcinoma

**DOI:** 10.1007/s12253-018-0475-8

**Published:** 2018-10-29

**Authors:** Ulana Kotowski, Lorenz Kadletz, Sven Schneider, Felicitas Oberndorfer, Boban M. Erovic, Matthaeus Ch. Grasl, Claudia Lill, Gregor Heiduschka

**Affiliations:** 1 Department of Otorhinolaryngology, Head and Neck Surgery, Medical University of Vienna, Waehringer Guertel 18-20, 1090 Vienna, Austria; 2 Department of Pathology, Medical University of Vienna, Waehringer Guertel 18-20, 1090 Vienna, Austria

**Keywords:** ELMO3, Molecular prognostic marker, Minor salivary gland cancer, Immunohistochemistry

## Abstract

Engulfment and cell motility 3 protein (ELMO3) is a protein that is involved in cell migration and promotes the remodeling of the cytoskeleton. Moreover, it is described as a prognostic marker in several cancers. The aim of this study was to evaluate ELMO3 expression in patients with minor salivary gland carcinoma. The expression of ELMO3 was examined by immunohistochemistry. The intensity of staining was evaluated and data was correlated to clinical outcome. Forty-six patients with complete clinical data were included into statistical analysis. ELMO3 expression was observed in 85% of the cases. High staining intensity of ELMO3 correlated with a significantly worse disease free survival (*p* = .0495) and a higher recurrence rate (*p* = .0071). In conclusion, it is still difficult to predict the clinical outcome of patients with minor salivary gland carcinoma. Evaluation of ELMO3 might serve as a clinical prognostic marker in future.

## Introduction

Minor salivary gland carcinomas are rare and derive from minor salivary glands located in the sinonasal cavities, oropharynx, larynx and trachea with the majority being found in the oral cavity [1]. The WHO (world health organization) classification currently describes about 24 different malignant epithelial tumors of the salivary glands, each with different biological behavior [2]. The cornerstone of treatment is complete surgical resection followed by radiotherapy with 5-year survival rates ranging from 66 to 80% [3–5]. However, due to the rarity and heterogeneity of this malignant disease, it is difficult to predict the clinical outcome of patients with minor salivary gland carcinomas. Clinical prognostic factors include tumor size, grade, surgical margins and lymph node status [6, 7]. However, to improve treatment outcomes a better understanding of molecular mechanisms involved in tumor biology is needed. This implies to establish diagnostic, prognostic and predictive markers that could potentially be targeted therapeutically as well [8].

ELMO3, a protein belonging to the engulfment and cell motility (ELMO) family, is involved in cell migration and promotes cytoskeletal remodeling [9]. Recently its prognostic significance in non-small lung carcinoma was demonstrated [10]. In particular, ELMO3 is significantly higher expressed in tumors from patients with distant metastases compared to normal lung tissue and to tumors from metastasis-free patients [11]. Furthermore, investigations in head and neck squamous cell carcinomas showed that ELMO3 expression correlates with a decreased overall survival (OS) and disease-free survival (DFS) [12]. A study in early glottic laryngeal cancer reported that expression of ELMO3 correlates with a poor OS and DFS as well [13]. The hypothesis is that during cancer progression the promoter region of ELMO3 is demethylated which is associated with the formation of metastasis. In contrast, promoter methylation in non-malignant cells results in transcriptional silencing [14]. Studies in human intestinal cancer cell lines showed that homeobox protein CDX2 plays a role in the transcriptional regulation of intestine-specific expression of ELMO3 [15].

The expression of ELMO3 in malignant tumors of minor salivary glands has not been studied so far. The aim of this study was to investigate the expression of ELMO3 in patients with minor salivary gland carcinoma using immunohistochemical staining. ELMO3 expression was correlated with patient data to evaluate a possible connection with survival.

## Materials and Methods

### Patients

Fifty patients with newly diagnosed carcinoma of the minor salivary glands, treated between 1974 and 2013 at the Medical University of Vienna, were included in this retrospective study. Clinicopathologic information was derived from medical charts and patients were classified according to the TNM classification of the Union for International Cancer Control (UICC). Patients that were lost to follow up (1 patient) or patients with missing tissue samples (3 patients) were excluded from statistical analysis. Subsequently, 46 patients were eligible for further investigations. The detailed patients data and histological classification are summarized in Table 1. This study was approved by the institutional ethics committee (EK 1926/2015). Informed consent was obtained from all patients and the study was performed in accordance with the Declaration of Helsinki.

**Table 1:** Clinical data for patients included in statistical analysis

Pat. no.	Localization	Histology	T	N	M	ELMO3 expression
1	Hard palate	ACC	T4	N0	M0	high
2	Hard palate	ACC	T2	N0	M0	high
3	Paranasal sinus	ACC	T2	N0	M0	high
4	Retromolar trigonum	ACC	T1	N0	M0	high
5	Hard palate	BCC	T1	N0	M0	high
6	Maxilla	ACC	T4	N0	M0	high
7	Tongue base	ACC	T3	N0	M0	low
8	Maxilla	CA ex PL	T3	N0	M0	high
9	Hard palate	MEC, lg	T2	N0	M0	high
10	Maxilla	CCC	T2	N0	M0	high
11	Hard palate	CA ex PL	T2	N0	M0	high
12	Paranasal sinus	ACC	T1	N0	M0	high
13	Maxilla	ACC	T1	N0	M0	high
14	Hard palate	ACC	T1	N0	M0	high
15	Paranasal sinus	ACC	T4	N0	M0	high
16	Soft palate	Adeno CA	T2	N0	M0	high
17	Paranasal sinus	ACC	T2	N0	M0	high
18	Upper lip	ACC	T1	N0	M0	high
19	Tongue	ACC	T1	N0	M0	high
20	Paranasal sinus	ACC	T3	N0	M0	low
21	Tongue base	ACC	T3	N0	M0	low
22	Paranasal sinus	CA ex PL	T3	N0	M0	low
23	Paranasal sinus	ACC	T3	N0	M0	low
24	Hard palate	CA ex PL	T2	N0	M0	low
25	Larynx	MEC, img	T2	N0	M0	low
26	Tongue base	MEC, lg	T2	N0	M0	low
27	Soft palate	ACC	T2	N0	M0	low
28	Mouth base	Adeno CA	T2	N0	M0	low
29	Hard palate	Adeno CA	T2	N0	M0	low
30	Tongue base	ACC	T1	N0	M1	low
31	Paranasal sinus	ACC	T1	N0	M0	low
32	Palate	MEC, lg	T1	N0	M0	low
33	Cheek	MEC, lg	T1	N1	M0	low
34	Tongue base	MEC, lg	T1	N1	M0	low
35	Paranasal sinus	Adeno CA	T4	N2b	M0	high
36	Mouth base	ACC	T4	N2b	M0	low
37	Cheek	ACC	T3	N0	M0	high
38	Auditory canal	Adeno CA	T1	N0	M0	high
39	Cheek	ACC	T1	N0	M0	low
40	Retromolar trigonum	ACC	T1	N0	M0	low
41	Larynx	Adeno CA	T2	N0	M0	low
42	Maxilla	ACC	T4	N0	M1	low
43	Larynx	ACC	T1	N0	M0	low
44	Retromolar trigonum	MEC, img	T1	N0	M0	low
45	Palate	MEC, lg	T1	N0	M0	low
46	Maxilla	MEC, lg	T4	N0	M0	high

*Pat. no.*, patient number; *ELMO3*, engulfment and cell motility 3 protein; *ACC*, adenoid cystic carcinoma; *MEC*, mucoepidermoid carcinoma; *lg*, low grade; *img*, intermediate grade; *Adeno CA*, adenocarcinoma; *CA ex PL*, carcinoma ex pleomorphic adenoma; *BCC*, basal cell adenocarcinoma; *CCC*, clear cell carcinoma

### Immunohistochemistry

Immunohistochemical staining was performed as described elsewhere using the Lab Vision Ultra V Block kit (Thermo Fisher Scientific, Waltham, MA, USA) and the Lab Vision Ultravision LP detection system (Thermo Fisher Scientific, Waltham, MA, USA) according to manufacturers protocol [16]. Briefly, citrate buffer (pH 6.0) was used for antigen retrieval. Anti-ELMO3 antibody (Sigma-Aldrich, St.Louis, MO, USA) was diluted to 1:500. Slides were incubated for one hour at room temperature. Paraffin-embedded samples of non-small-cell lung carcinoma and squamous cell carcinoma served as positive control. For negative control primary antibody was replaced by rabbit immunoglobulin G isotype control (Abcam, Cambridge, United Kingdom). Samples were analyzed using an Olympus BH-2 microscope (Olympus, Tokyo, Japan). Based on the intensity of the cytoplasmic staining of neoplastic cells, all samples were assigned to one of four categories: 0: negative; 1: weak; 2: moderate; 3: strong. The assignment was performed by two independent investigators (FO and LK). Subsequently, the percentage of stained neoplastic cells was taken into account (<10% negative; >10% positive) and specimens were characterized as ELMO3 positive or ELMO3 negative. For statistical analysis we combined patients with negative and weak expression of ELMO3 to the group of “ELMO3 low” patients and patients with moderate and strong expression to the group of “ELMO3 high” patients.

### Statistical Analysis

For clinical and patients’ data, we used descriptive statistics. Additionally, Fisher’s exact test was used to compare categorical data between two groups and chi square test was used in case of 3 or more groups. Rates of OS and DFS were calculated by means of the Kaplan-Meier method. We used the log-rank test (Mantel-Cox) to assess statistical differences between the established patient groups**.** Furthermore, hazard ratios (HR) were calculated and according 95% confidence intervals (CI) were established to reflect a significance level of 0.05. Moreover, 2 × 2 tables with incidence data of death and recurrence were constructed and analyzed by using Fisher’s exact test. Multivariate analyses were performed using the Cox regression model in order to identify independent variables. Thereby all variables were included into multivariate analysis. Statistical significance was determined as *p* < .05. SPSS software version 21.0 (SPSS Inc., Chicago, IL) and Prism Graphpad (Graphpad Software Inc., La Jolla, CA) were used to analyze and visualize the data.

## Results

### Clinical Data

Forty-six patients with complete medical data were stained for ELMO3 expression. Out of them, 25 (54.3%) patients were diagnosed with an adenoid cystic carcinoma, 9 (19.6%) patients had a mucoepidermoid carcinoma, 6 (13%) patients had an adenocarcinoma, 4 (8.7%) patients had a carcinoma ex pleomorphic adenoma, and in one (2.2%) patient each basal cell adenocarcinoma and clear cell carcinoma was found. Of these patients, 22 were male (48%) and 24 were female (52%). The mean age was 57 years (range 25–88 years, median 61 years). At time of diagnosis 18 (39.1%) patients presented with T1 disease, 14 (30.4%) patients had a T2 classification and 7 (15.2%) patients each were diagnosed with T3 and T4. Forty-two patients (91.3%) did not show any lymph node metastases and were therefore classified as N0. Four patients (8.6%) presented themselves with N2b classification. Two patients (4.3%) had distant metastases. All patients received either surgical resection with or without irradiation, depending on the staging, or radiochemotherapy. Of the patients with surgical therapy, four had a R1 resection. The mean follow-up period was 5.3 years (range 0.2–31.6 years). Next, rates of 10-year survival were calculated for our cohort. Median OS measured 6.3 years and median DFS was calculated as 5.0 years. A total of 27 (58.7%) patients developed recurrent disease and 23 patients (50%) died during the observation period.

### Expression of Engulfment and Cell Motility 3 in Malignant Tumors of the Minor Salivary Glands

**Fig. 1:**
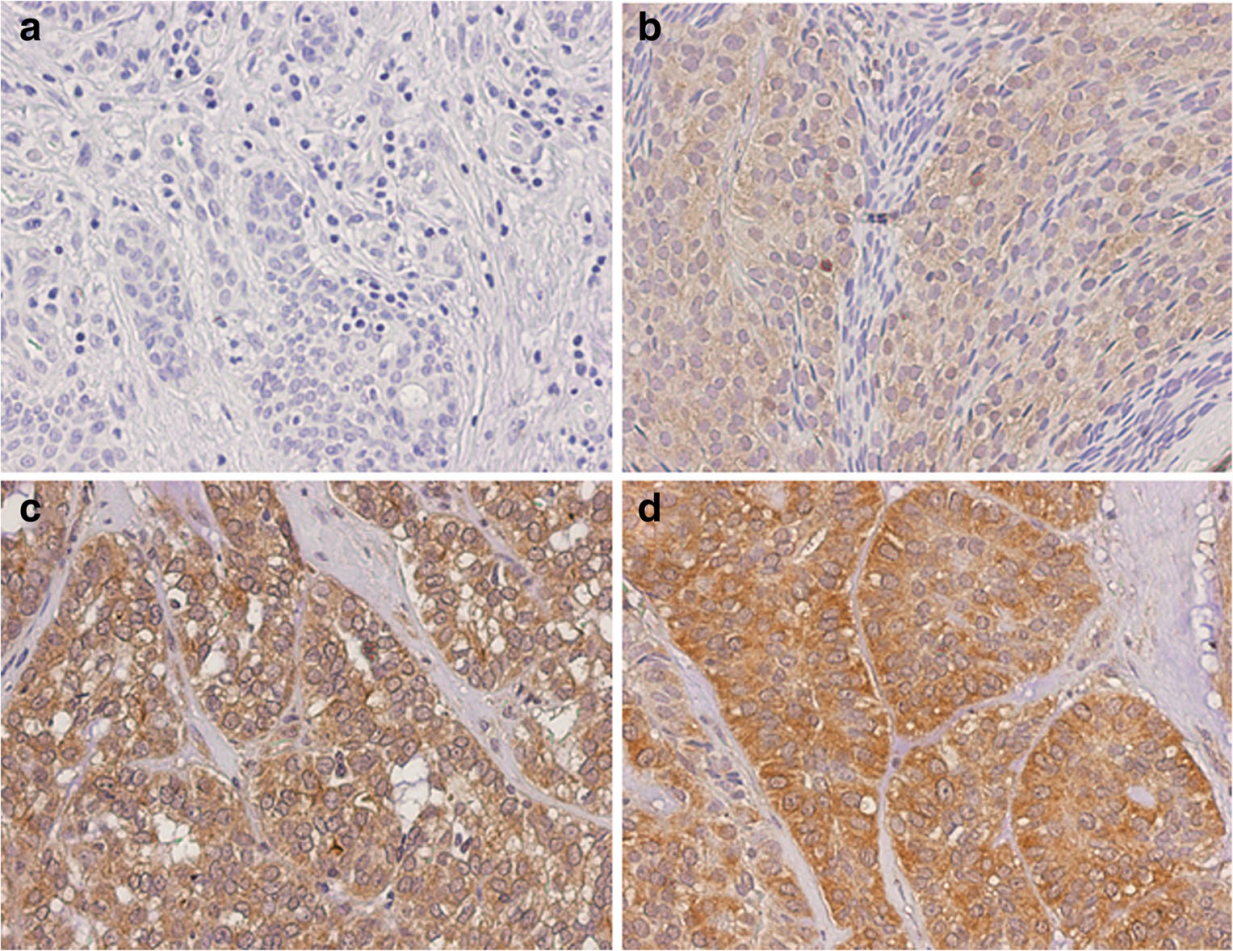
Immunohistochemical expression analysis of ELMO3 in samples from patients with minor salivary gland carcinoma. Examples of tumors with (**a**) no, (**b**) weak, (**c**) moderate and (**d**) strong ELMO3 expression

All samples were analyzed independently by two examiners. The calculated kappa value was 0.652. ELMO3 expression was detected in 39 (85%) of 46 samples. The expression of ELMO3 could always be found in the cytoplasm of the cancer cells (Fig. 1). Weak expression was found in 17 (37%), moderate expression in 15 (32.6%) and strong expression was found in 7 (15.2%) of the analyzed samples. Seven (15.2%) samples showed no staining. Adjacent connective tissue, nerves, and vessels showed no staining. Inflammatory cells showed a weak to focal moderate staining. For further statistical analysis the groups “no expression” and “weak expression” were summarized to an “ELMO3 low” staining group. “Moderate expression” and “strong expression” were summarized to an “ELMO3 high” staining group.

### Evaluation of Expression Patterns Stratified by Histological Classification

*Adenoid cystic carcinoma:* In adenoid cystic carcinoma, 13 (52%) of 25 patients had a high ELMO3 staining and 12 (48%) patients had a low ELMO3 staining.

*Mucoepidermoid carcinoma:* High ELMO3 expression was detected in two (28.6%) of seven low grade mucoepidermoid carcinoma samples and low ELMO3 expression in five (71.4%) of these samples. In intermediate grade mucoepidermoid carcinoma samples two (100%) of two samples showed low ELMO3 expression.

*Adenocarcinoma:* In adenocarcinoma 50% (three of six samples) of the samples showed high ELMO3 expression and 50% low ELMO3 expression.

*Carcinoma ex pleomorphic adenoma:* In patients with carcinoma ex pleomorphic adenoma 50% (two of four samples) showed high ELMO3 expression and 50% showed low ELMO3 expression.

*Basal cell adenocarcinoma:* In basal cell adenocarcinoma 100% (one of one) of the samples showed high ELMO3 staining.

*Clear cell carcinoma:* High ELMO3 staining was detected in 100% (one of one) of the patients with clear cell carcinoma.

### Correlation between Engulfment and Cell Motility 3 Expression and Clinicopathological Characteristics

**Fig. 2:**
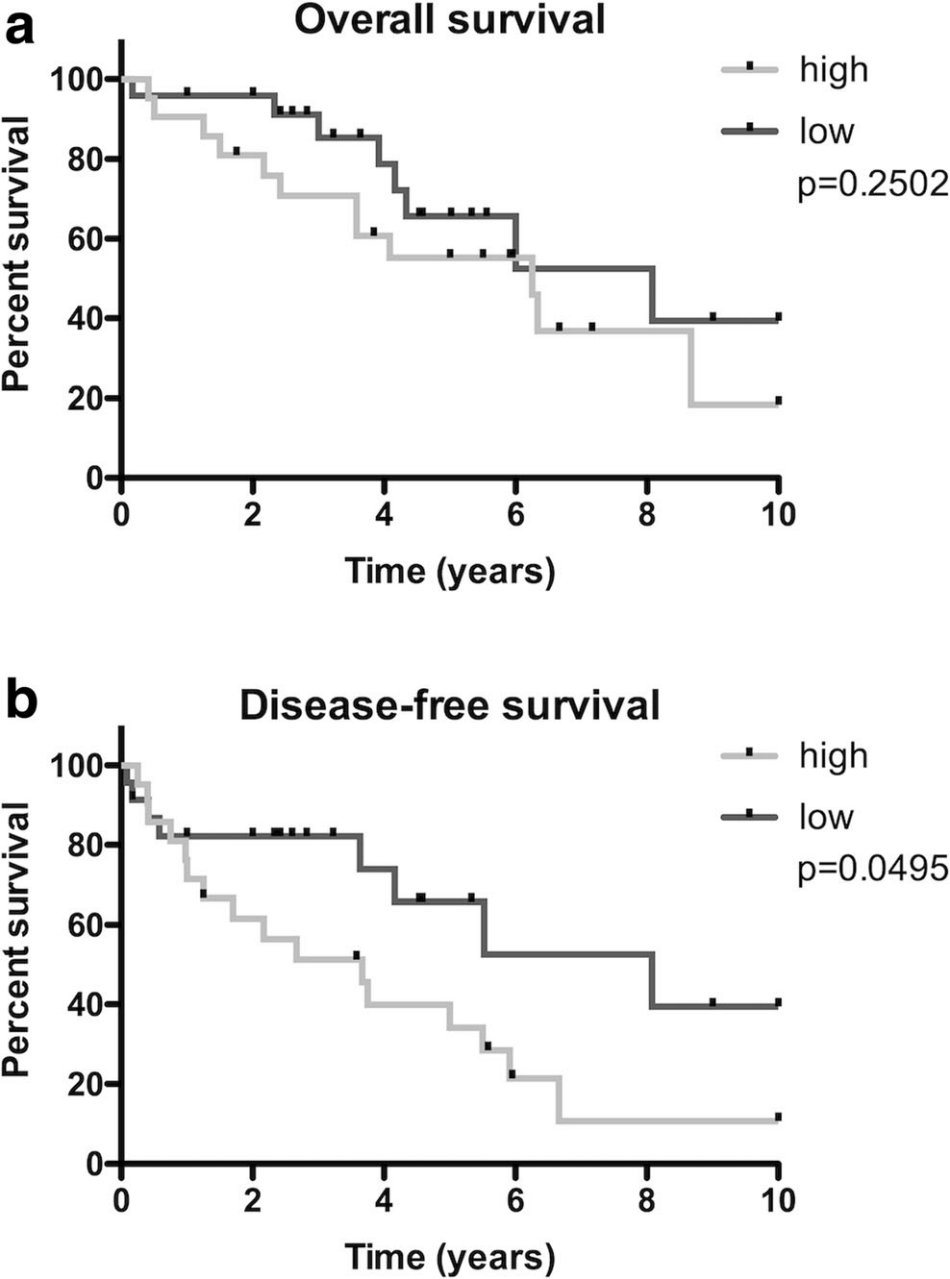
Kaplan-Meier estimates for (**a**) overall survival (OS) and (**b**) disease-free survival (DFS) according to ELMO3 expression intensity in patients with minor salivary gland cancer

**Fig. 3:**
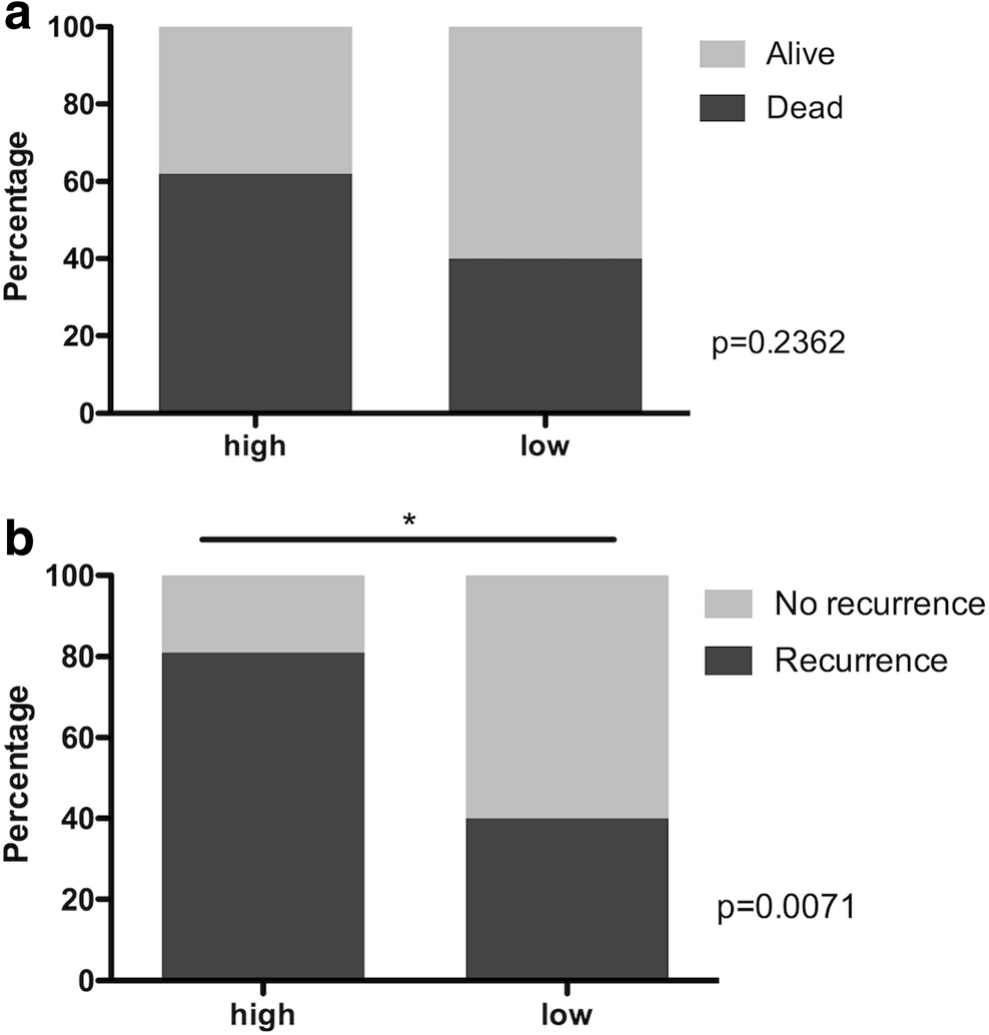
(**a**) Survival and (**b**) recurrence rates after total follow up according to ELMO3 expression intensity in patients with minor salivary gland cancer

OS and DFS were calculated. The OS rate after 10 years was 39.4% for patients with low ELMO3 expression and 18.4% for patients with high ELMO3 expression (Table 2). These results were not statistically significant (*p* = .2502). However, in terms of DFS, a significantly worse outcome for patients with high ELMO3 expression was observed. DFS after 10 years was 39.4% for patients with low ELMO3 expression and 10.7% for patients with high ELMO3 expression (*p* = .0495) (Fig. 2). Moreover, patients with high ELMO3 expression showed a significantly increased recurrence rate (*p* = .0071). After total follow up, 80% of the patients with high ELMO3 staining had a recurrence as compared to 40% of patients with low ELMO3 staining (Fig. 3). Regarding the OS, there was no statistically significant difference between the high and low staining groups. However, more patients with high ELMO3 staining died compared to the low staining ELMO3 group (*p* = .2362).

**Table 2:** Statistical analysis of patients with minor salivary gland cancer depending on ELMO3 expression intensity

	ELMO3 high	ELMO3 low	
Overall Survival			*p* = .2502 HR 1.68 (CI 95% 0.69–4.07)
2-year survival	80.9%	95.8%	
5-year survival	55.2%	72.2%	
10-year survival	18.4%	39.4%	
Median survival	6.25 years	8.08 years	
Disease-free Survival			*p* = .0495 HR 2.20 (CI 95% 1.01–5.05)
2-year survival	61.5%	82.2%	
5-year survival	34.2%	65.7%	
10-year survival	10.7%	39.4%	
Median survival	3.66 years	8.08 years	

*ELMO3*, engulfment and cell motility 3 protein; *HR*, hazard ratio; *CI*, confidence intervall

Moreover, univariate and multivariate analyses were performed to evaluate if ELMO3 expression serves as an independent marker for DFS. ELMO3 expression, T classification, N classification, histological classification, tumor localization and resection margins were included into calculation. Since malignancies of the minor salivary glands located in the nasal cavity and/or paranasal sinuses have a significantly worse survival [17], tumors in this localization were compared to tumors in the remaining localizations. As aforementioned univariate analysis showed that ELMO3 is a negative prognostic factor for DFS. Beside ELMO3 expression, adenoid cystic carcinoma (*p* = .033) and adenocarcinoma (*p* = .018) were associated with reduced DFS rates in univariate analysis. However, multivariate analysis revealed that DFS is significantly depending on the histologic subtype (Table 3).

**Table 3:** Multivariate analysis of disease-free survival

Variable	Exp (B)	95.0% CI for Exp (B)		*p* value
		Lower	Upper	
ELMO3	2.218	0.788	6.247	.132
T	1.038	0.682	1.579	.863
N	1.813	0.621	5.293	.276
Histology	0.670	0.467	0.960	.029
Nasal cavity/paranasal sinuses	0.875	0.305	2.511	.804
R1 Resection	0.430	0.108	1.707	.230

*ELMO3*, engulfment and cell motility 3 protein; *Exp (B)*, hazard ratio; *CI*, confidence interval

## Discussion

Minor salivary gland carcinoma is a rare tumor and little is known about the underlying molecular biologic processes in this type of cancer. The world health organization (WHO) classifies 24 types of malignant epithelial tumors of the salivary glands with adenoid cystic carcinoma and mucoepidermoid carcinoma being the most frequent pathologies [3]. In our cohort 54.3% of the patients had an adenoid cystic carcinoma and 19.6% of the patients had a mucoepidermoid carcinoma indicating that a representative group of patients has been evaluated. As prognostic factors for patients outcome, tumor size, histological grade, lymph node metastasis, surgical resection margins and perineureal invasion have been described [7, 18, 19]. However molecular prognostic factors are required to further stratify the patients. In this study the expression pattern of ELMO3 in minor salivary gland carcinoma has been investigated. Furthermore, presence of ELMO3 was correlated with survival data.

Recently, ELMO3 has been described as a negative prognostic biomarker in non-small cell lung cancer [10], head and neck squamous carcinoma [12] and T1 laryngeal cancer [13]. In vitro, ELMO3 was also detected in human intestinal cancer cell lines [15]. To the best of our knowledge, this is the first study assessing the expression of ELMO3 in minor salivary gland carcinoma. In our cohort, an expression of ELMO3 was detected in 85% of the patients. Similarly, ELMO3 expression was detected in 71.2% of head and neck squamous cell squamous carcinoma cases as reported by Kadletz et al. [12]. In contrast, ELMO3 expression in early glottic cancer was only found in 23% of the patients [13].

Furthermore, we found a statistically significant negative effect of high ELMO3 expression in terms of DFS in patients with minor salivary gland carcinoma. Patients with high ELMO3 expression had a DFS of 10.3% after 10 years, whereas patients with low ELMO3 expression showed a DFS of 39.4% after 10 years. These results are in accordance with findings in head and neck cancer [12, 13]. Studies in non-small cell lung cancer tumors showed that ELMO3 expression is higher in patients with metastasis than in normal lung tissue and patients without metastasis indicating a poor prognosis for patients with high ELMO3 expression [9].

Moreover, recurrence rate was significantly higher in patients with high ELMO3 staining intensity (80%) compared to patients with low staining intensity (40%), which underlies the hypothesis that ELMO3 expression goes along with a poor outcome.

We are aware of the limitations of the study due to the heterogeneity of the investigated samples. However, small salivary cell carcinoma is a rare disease and our cohort contained a representative group of patients.

In conclusion, we could demonstrate that overexpression of ELMO3 is associated with shortened DFS and higher risk for recurrent disease. Therefore we think that ELMO3 might serve as a negative prognostic marker in patients with minor salivary gland cancer.
